# Correction to “Prevention and treatment of venous thromboembolism in patients with multiple myeloma: Clinical practice guidelines on behalf of the European Myeloma Network”

**DOI:** 10.1002/hem3.70250

**Published:** 2025-11-14

Gerotziafas G, Fotiou D, Nijhof I, et al. Prevention and treatment of venous thromboembolism in patients with multiple myeloma: clinical practice guidelines on behalf of the European Myeloma Network. *HemaSphere*. 2025;9(8):e70177. doi:10.1002/hem3.70177

In the author listing of the manuscript, the name of an author was incorrectly listed as Alessandra Laroca. The correct name is Alessandra Larocca.

The original publication has been corrected. We apologize for this error.

